# Solution-focused brief therapy for common mental disorders in the light of empirically supported treatment revised criteria: a systematic review protocol

**DOI:** 10.3389/fpsyt.2025.1602060

**Published:** 2025-11-11

**Authors:** Krzysztof Pękala, Michał Seweryn, Andreea M. Żak

**Affiliations:** 1 Department of Medical Psychology, Medical University of Lodz, Lodz, Poland; 2 Centrum Terapii Krótkoterminowej, Lodz, Poland; 3 Center for Digital Biology and Biomedicine, University of Lodz, Lodz, Poland; 4 Regional Digital Medicine Center, Copernicus Memorial Cancer Center, Lodz, Poland; 5 ”Differently” Psychotherapy Center, Wadowice, Poland

**Keywords:** empirically supported treatment, solution-focused brief therapy, efficacy, effectiveness, common mental disorders, systematic review protocol

## Abstract

**Background:**

Solution-Focused Brief Therapy (SFBT) is a short-term, goal-directed therapeutic approach widely used across diverse settings. While prior evidence supports its overall effectiveness, the empirical status of SFBT as a treatment for common mental disorders (CMD) remains unclear. This protocol outlines a systematic review aiming to evaluate the efficacy, effectiveness, mechanisms of change, and cost-effectiveness of SFBT in the treatment of CMD following the updated empirically supported treatment criteria.

**Methods:**

A systematic search for recently published systematic reviews, randomized controlled trials, and high-quality non-randomized studies of intervention examining SFBT in adults (≥18 years) diagnosed with CMDs will be performed. Risk of bias and methodological quality will be assessed. Data selection, extraction, and rating will be conducted independently by at least two reviewers.

**Outcomes:**

Primary outcomes include symptom reduction in CMD; secondary outcomes encompass, among other improvements in psychosocial functioning and remission rates. Additional outcomes involve cost-effectiveness, adverse effects, and evidence for mechanisms of change. Meta-analysis and narrative synthesis will be performed when appropriate.

**Expected Impact:**

The rigorous assessment of SFBT empirical status in the context of CMD can potentially influence recommendations for clinical practice, guideline development, and future research.

## 1 Introduction

Solution-Focused Brief Therapy (SFBT) is a short-term, goal-and-future-oriented, social constructionist therapeutic approach that emphasizes the client’s strengths and resources in building solutions rather than focusing on analyzing or resolving problems (1, 2). SFBT is widely used in diverse clinical and non-clinical settings with research evidence showing positive impact on various outcomes for diverse populations (3, 4). Nevertheless, a synthesis of evidence in the clinical setting following new proposed empirical support treatment (EST) guidelines (5) has not been yet produced, probably due to the paradigm’s non-diagnostic focus. Although SFBT is formulated around client-defined goals rather than diagnoses (1, 2), we structure this review by common mental disorder (CMD) categories for pragmatic reasons: (i) to maximize comparability of outcome measures and enable quantitative synthesis across studies; (ii) to align with how guidelines, payers and service planners appraise evidence; and (iii) to facilitate knowledge translation to non-SFBT clinicians. We retain the transdiagnostic emphasis by accounting also for SFBT-specific indicators such as goal-attainment or perception of self-efficacy alongside improvements in functioning as important outcomes. The proposed systematic review aims to assess SFBT’s efficacy, effectiveness, cost-effectiveness, and mechanisms of change in the context of CMDs in line with a new EST guideline (5).

Historically, the field of psychotherapy has long been concerned with the question of effectiveness which is still actual: “What treatment, by whom, is most effective for this individual with what specific problem, under which set of circumstances, and how does it come about?” (6). To answer to this lingering question the evidence-based practice in psychology (EBPP) was structured around the same methodological principles used to test pharmacotherapy. Randomized controlled trials (RCT) were proposed as a golden standard of methodology (7), despite obvious applicability issues in the psychotherapeutic context (5, 8, 9). Just to mention one, neither participants or therapists can be fully blinded to the intervention as it can happen in the pharmacotherapeutic context. The overreliance on RCT moved the focus from treatment effectiveness to efficacy under controlled conditions and strict protocols, leading to the reliance of EBPP on artificial treatments applied in artificial contexts by an unreal specialist to an unreal individual (9). The evaluation criteria undergo constant discussions and adaptations to fit the psychotherapeutic context. Current proposals put accent on three components of EBPP with the best available research evidence (reflected in EST) as one of the main components alongside therapists’ clinical expertise and patients’ characteristics and preferences (10). In this framework EST represents the basis for clinical judgment when deciding which treatment may fit better to which client by accounting for individual and contextual characteristics (10).

The new approaches on EST (5, 8, 11, 12) go beyond the overreliance on studies performed in controlled conditions by including additionally research performed in the naturalistic setting where real life practice takes place. Treatment protocols are currently considered as open guidelines adjustable during the therapeutic process in line with patients’ needs and based on the professional’s clinical expertise. Assessment measurements go beyond quantitative measures and include qualitative ones concerned with the individual’s perception of treatment impact. Treatment effectiveness is measured by accounting for additional outcomes, such as psychosocial functioning, rather than just symptom reduction despite the latter still regarded as the primary one (5, 8). Greater reliability is given to high-quality systematic reviews to compensate for potential risk of bias included in individual studies, be they RCT.

Among existing proposals, we chose to follow Tolin’s et al. (5) updated EST recommendations, due to their comprehensiveness in addressing all these above-mentioned aspects, being also adopted and promoted in research meant to impact future recommendation lists of psychological treatments for CMDs (13). The position of an approach within the clinical setting may thus be influenced by how empirical evidence fits these new EST criteria; unverified approaches risk being left behind. Assessment is based on the treatment’s effect on symptoms and individuals’ functioning, with maintenance for at least 3-months after the intervention. Recommendations categories (very strong, strong, or weak) are based on the quality of evidence rated by the Grading of Recommendations, Assessment, Development, and Evaluation (GRADE) system (14, 15). Priority is given to recent high-quality systematic reviews (preferably published within the last two-years) over single study tallies, accounting for treatment fidelity and contextual factors. Aligned with EBPP, the EST recommendations highlight the integration of best current evidence, clinician expertise, and patient values, by considering patient-relevant outcomes (functioning) alongside clinical-setting relevant ones (symptom change) with planned analyses of adverse events, cost-effectiveness, and mechanisms of change.

The mechanism of change in SFBT is centered around the co-construction of the client’s preferred future, existing strengths and resources, and observable micro-changes identified through analyses of progress or exceptions (instances when the problem is less intense or absent) (1, 2). In line with the main assumptions, the process of solution-building differs from problem-solving, and thus the focus goes beyond symptom modification. Clients are assumed to already possess solution-building capacities, the therapeutic process focuses on identifying and amplifying them. The therapist’s language has a central role, by intentional use of presuppositions of change while constructing each statement based on the client’s words (1, 2). Main techniques include: (i) goal negotiation and preferred-future descriptions (clarifying what the client expects to be different, presupposing that the therapy is helpful and describing in details the preferred future presupposing the fulfillment of the expectations), (ii) exception-eliciting questions followed by an amplification of what already works, (iii) scaling questions to track progress, confidence, and motivation towards change, and (iv) resource amplification and compliments to bring strengths into awareness and consolidate their use. Additionally, sessions can end with a structured end-of-session feedback with concrete between-session tasks. A meta-analysis of process-outcome studies (16) confirmed the relevancy of the proposed set of interventions including the specific use of language which was extensively examined in microanalysis research (17, 18).

Being a psychotherapeutic approach, the effect of SFBT was researched in relation to various internalizing, externalizing, school, social, work or couple issues. While such evidence is not needed by or in line with the theoretical background of SFBT, it has the potential to facilitate communication with experts from the medical, psychological, administrative and political field which still tend to use diagnostic categories as a frame of intervention. Many systematic reviews were already conducted, with findings consistently indicating a predominance of positive outcomes (3, 4). Clients reported significantly better results compared to no treatment or similar results to other well-known treatments for a wide variety of emotional, behavioral, relational or functional outcomes. Better results were observed in non-randomized naturalistic studies than in controlled conditions especially among individuals without a formal diagnosis (3). This difference may be due to treatment modality, as individual intervention specific to the clinical setting was associated with lower effects compared to group intervention specific to non-clinical setting (4). Nevertheless, a detailed examination of SFBT effect and mechanism of change in the context of CMDs is yet to be performed.

## 2 Objectives

The aim of the currently proposed systematic review is to evaluate the empirical support of SFBT for CMDs in line with Tolin et al.’s (5) criteria for EST. Specifically, we plan to systematically review and evaluate:

- the clinical meaningful effect of SFBT on CMDs symptoms and its maintenance for at least 3-months after treatment;
- the clinical meaningful effect of SFBT on psychosocial functioning of individuals diagnosed with CMD and its maintenance for at least 3-months after treatment;
- the mechanisms of change and key therapeutic factors contributing to SFBT outcomes;
- the cost-effectiveness of SFBT and potential adverse effects.

Based on the findings we plan to make proposals for clinical interventions, identify gaps in the current literature, and recommend directions for future research.

### 2.1 Review questions

The proposed systematic review plans to answer to the following questions:

- What is the efficacy and effectiveness of SFBT in the context of CMDs compared to passive or active control conditions, including comparison to other known psychotherapeutic treatments? Specifically (i) What is the efficacy and effectiveness of SFBT on CMDs symptom reduction compared to control or comparison groups?, and (ii) What is the efficacy and effectiveness of SFBT on psychosocial functioning, quality of life or other positive outcomes for individuals diagnosed with CMDs compared to control or comparison groups?
- What are the after-treatment effects of SFBT on CMDs symptoms, and individuals psychosocial functioning?
- What mechanisms of change underpin the therapeutic outcomes of SFBT?
- Is SFBT a cost-effective intervention for CMDs?

## 3 Methods

### 3.1 Eligibility criteria

#### 3.1.1 Types of studies

High quality systematic reviews, meta-analyses, and RCTs comprising outcome or process-outcome studies will be included to show the efficacy of SFBT for specific CMDs. Additionally, NRSI examining mechanisms of change, effectiveness in real-world settings, or cost-effectiveness will be included to provide an image of the effectiveness outside the controlled settings of RCT. All other types of studies will be excluded. Publications will be considered with no language restrictions.

The quality of the studies will be assessed as follows:

- systematic reviews will be assessed using AMSTAR 2 (19),
- individual studies’ risk of bias will be assessed using the revised version of the Cochrane Risk of Bias Tool for RCTs (20), respectively ROBINS-I for NRSI (21).

Primary studies will be included only if minimal bias is found in relevant domains such as confounding, selective reporting and outcome measurement. Nevertheless, considering previous evidence using AMSTAR 2 to assess the quality of systematic reviews of psychological interventions (4, 22), we expect to find some critical flaws in those studies. Depending on the type and number of identified flaws a discussion will be held in the review panel to decide the inclusion or exclusion of each individual systematic review. Furthermore, if a sufficient number of high-quality primary studies are not identified, i.e., minimum three different studies for the same specific outcome, we will also include studies of moderate quality, ensuring that conclusions are drawn with careful consideration of their limitations. Primary studies of low quality will be excluded. Next, the strength of recommendations and quality of evidence will be assessed using GRADE (14, 15).

All ratings will be conducted independently by two raters, with any discrepancies resolved through consensus or, if necessary, by involving a third rater.

#### 3.1.2 Population

The population will be restricted to adults (≥18 years) diagnosed with CMDs or presenting specific clinical symptoms. Studies performed on mixed age samples not providing separate results for the adult population will be excluded.

Adults diagnosed with CMDs, as classified by the DSM-5 or ICD-10 (excluding organic mental disorders), will be considered. These include:

- Depressive disorders,
- Anxiety disorders,
- Trauma- and stressor-related disorders,
- Dissociative disorders,
- Obsessive-compulsive disorders,
- Eating disorders,
- Somatic symptom disorders,
- Attention deficit/hyperactivity disorder (ADHD),
- Substance use disorders,
- Personality disorders including specific types of disorders,
- Bipolar disorders,
- Schizophrenia spectrum disorders.

Since mental disorders rarely occur in isolation, we will, where reported, code comorbidity as a dummy variable (present/absent) to examine whether and how efficacy and effectiveness vary. Nevertheless, considering our focus on decrease in symptoms severity and improvement in functioning outcomes, we will not perform stratified analyses (within diagnosis).

#### 3.1.3 Intervention

To be included, studies should explicitly use SFBT as a sole intervention, implementing its active ingredients such as: goal setting, future-orientation, co-construction using clients’ language and focus on progress and clients’ strengths. Only studies which provide a clear description of the intervention or declaration of adherence to treatment by following a manual or providing evidence of training and practice experience will be included. Considering that some degree of heterogeneity of the intervention is normally expected between studies (5), efforts will be made to ensure commonality, i.e., the presence of the same core elements and assumptions, to allow the integration of diverse studies into a cohesive analysis of a unified approach. For this purpose, we will use as reference the newest edition of the SFBT treatment manual (23), due to its root in the core assumptions and active ingredients promoted by the founders of the approach while including also current advancements in the field based on empirical evidence.

To ensure high fidelity to SFBT, only studies using at least four core elements will be considered for selection, in line with the recommendations from previous systematic reviews (3, 16). We guided the selection of the minimal core elements based on existing theoretical arguments (24), empirical evidence (16, 25), and the newest treatment manual (23). Thus, the minimal elements presented in a primary study to be included are (i) the specific use of language during the co-construction of meaning, (ii) goal definition by having a description of the preferred future, (iii) examination of previous instances where the solution was already present (exceptions to the problem), and (iv) progress towards the goal (by using scaling questions and/or setting next steps).

The mode of delivery (individual vs group or family) will be assessed considering its confirmed relevance on SFBT outcomes (3, 4).

#### 3.1.4 Comparators

We will consider both active controls such as other known approaches (e.g., cognitive-behavioral therapy, psychodynamic), treatment-as-usual (TAU), pharmacotherapy or placebo interventions, and passive controls such as no treatment groups or waitlist.

#### 3.1.5 Outcomes

Three types of outcomes will be considered in line with their relevance in the mental health care (8, 26) which aligns with the chosen EST criteria (5):

- primary, i.e., symptom reduction assessed by validated scales. Two cases are to be discriminated here: (i) the binary outcomes represented by any decrease in the severity of a given symptom, and (ii) the magnitude in the decrease of the given symptom measured on defined scales;
- secondary, i.e., improvements in psychosocial functioning, alongside SFBT-specific outcomes such as treatment goal-attainment or self-efficacy by rating scales accounting also participants’ subjective evaluation;
- additional, i.e., cost-effectiveness (the treatment cost shall be measured as number of sessions and duration divided by health effect measured as primary and secondary outcomes), adverse effect (other unwanted negative outcomes), and evidence for mechanisms of change (dependency between the use of a specific active ingredient and primary and secondary outcomes).

#### 3.1.6 Time and setting

Treatment (end of treatment outcomes) and maintenance (at least 3-months after treatment outcomes) effect will be considered. Two cases are to be discriminated here: (i) the binary outcomes represented by maintenance of change after treatment, and (ii) the time frame for which such maintenance is confirmed.

Outcomes will be organized by different settings such as inpatient (hospital), outpatient (ambulatory), and community/non-medical setting (educational, social services).

### 3.2 Search and selection strategy

A comprehensive search will be conducted in the Cochrane Database of Systematic Reviews, ERIC, Europe PubMed Central, PubMed, Embase, WorldWideScience, CNKI, the Iranian Scientific Information Database, and Google Scholar for systematic reviews, meta-analyses, RCTs, and NRSIs published from 2022 up to the present in line with the timeframe recommended by the new evaluation criteria which guide the current study (5). If evidence is insufficient to conduct analyses for a given CMD, we will extend the search back to 2015 to ensure adequate coverage while still retaining contemporary methods. Search terms will include ‘Solution-Focused Brief Therapy,’ ‘SFBT,’ ‘brief therapy,’ solution-focused approach,’ and ‘solution-focused therapy,’ combined with ‘meta-analysis,’ ‘systematic review,’ ‘randomized controlled trial,’ ‘efficacy,’ ‘effectiveness,’ ‘cost-effectiveness,’ ‘mechanisms of change,’ and common mental health diagnoses and clinical symptoms as classified in the ICD-10 or DSM-5 and described in the Population section.

A hand search of relevant journals and references lists from included studies will be complementary performed alongside the consultation of reference lists maintained by the European Brief Therapy Association (EBTA) and Solution-Focused Brief Therapy Association (SFBTA). Additionally, large language models (LLMs) connected to the internet may be used to support the search process by identifying potentially relevant studies and refining search strategies through natural language understanding.

Selection will be based on a checklist to ensure fit with the eligibility criteria and inclusion of key parameters such as adherence to SFBT principles, maintenance effect, generalizability, and mechanisms of change. Treatment fidelity will be assessed by evaluating adherence to manuals, therapist training and experience, and the empirical validation of intervention integrity, in accordance with predefined intervention criteria as described in the Intervention section.

Inter-rater agreement (Cohen’s κ) on data selection will be calculated.

### 3.3 Data extraction

Data will be extracted using a standardized pre-defined form, including:

- study authors, year of publication, design and type of control, research questions, data collection and data analysis methods, and quality assessment;
- population characteristics including demographic information such as country, age, gender and information about the diagnosis and setting where the study was performed;
- intervention details and fidelity methods ensuring adherence to SFBT principles.

Data extraction will be conducted separately by two of the authors (KP and AŻ), blindly. In case of missing data or need for additional clarification, the primary study’s authors will be contacted. Discrepancies will be documented in a log and resolved by consensus or (when necessary) will be adjudicated by a third author (MS). If additional clarification is required, the original authors will be contacted prior to final adjudication.

### 3.4 Data synthesis and analysis

Quantitative or qualitative data analyses will be performed depending on the available information. Specifically, the quantitative approach will be used when a clear scale on which a given symptom is measured can be defined. The qualitative approach will be used with the purpose to describe the nature of the relationship between the phenomena of interest.

#### 3.4.1 Quantitative analysis

Meta-analyses will be conducted where possible, using effect sizes and confidence intervals to compare SFBT with other active treatments or to no treatment. Heterogeneity will be assessed using for example the I² statistics. Publication bias will also be assessed using funnel plot test (27) or other suitable method. Next, the confidence in the quality of evidence and strength of treatment recommendations will be assessed following the GRADE guidelines (5, 14, 15).

We expect analyses to be possible for symptom reduction, improvement in functioning, and other relevant outcomes measured quantitatively. The reduction in symptoms severity will be measures as a binary outcome as well as an ordinal variable. Due to the overlap in the very definitions of the symptoms, we plan to use a multi-dimensional definition of the outcome(s) and propose an approach based on embeddings (using correspondence analysis or non-linear distance-based embeddings - UMAP, t-SNE). In such case, we will use the coordinates of the embeddings as the “new” outcomes. In this way, we may use the standard effect size measures like the Cohen’s (d, omega, h), Hedges and Olking coefficients, and where appropriate other standard measures. Where appropriate the ordinal regression will be used with the conditional probabilities of the symptom severity score exceeding a given value defined as the dependent variable.

#### 3.4.2 Narrative synthesis

A descriptive summary will be provided for studies where meta-analysis is not feasible, for example when qualitative data is provided for the outcomes of interest (e.g., individuals’ perception of symptom improvement or mechanism of change reflected in clients’ perception of the treatment). Qualitative themes will be formulated based on commonalities.

#### 3.4.3 Subgroup or subset analysis

Where applicable, findings from specific subgroups or subsets of the study population will be analyzed. We expect to find factors such as:

- duration of treatment or dosage: evaluating how different treatment lengths or dose levels influence outcomes;
- professionals’ characteristics: hours of training or years of experience in applying SFBT;
- participants’ characteristics: diagnostic category, age group, gender or other available demographic factors.

This approach ensures a more detailed understanding of variations in the results across different segments of the population or study characteristics.

### 3.5 Timeline

Search and Screening: Months 1-3.

Data Extraction and Quality Assessment: Months 4-6.

Data Synthesis and Drafting the Review: Months 7-9.

Submission for Publication: Month 12.

### 3.6 Adverse events

Systematic reviews and studies on potential harms or unintended effects of SFBT will be included to assess risk versus benefit.

### 3.7 Stakeholder involvement

The systematic review will be conducted by the authors of the current protocol, all with experience in conducting reviews, two of them (KP and AŻ) being certified psychotherapists in line with the national guidelines applying the solution-focused approach in their private practice. Both of them are trainers of the SFBT in contact with esteemed professionals regarded nationally or internationally as developers of the approach. If required, help and review from scientists and practitioners from different SFBT associations will be requested. In this regard we acknowledge a potential allegiance bias and will mitigate it via preregistration and dual blinded screening, with the involvement of author MS (not familiar with the SFBT) in the screening process as a judge in solving disagreements and testing the adequacy of selected research against the established framework. Specifically, a first pool of data selection will be performed by all three authors to test for potential selection bias. If significant disagreement is found based on authors familiarity with the model, then a fourth neutral screener will be involved, and selection will be made in pairs combining familiar with non-familiar screener. Additionally, to ensure neutrality, MS will be responsible for data analysis and will check the adequacy of the interpretation of the findings in line with the empirical data.

## 4 Anticipated impact and benefits

This systematic review will provide a comprehensive synthesis of the empirical evidence of SFBT for CMDs. It will inform clinical practice and contribute to guideline development by clarifying the treatment’s efficacy and effectiveness, mechanisms of change, and practical applications in the clinical setting. We expect these results to be of relevance to policy makers, interested in funding and promoting the best available support for specific clinical issues. Based on the findings we plan in the future to share a taxonomy for SFBT fidelity (principles ↔ elements matrix). While it is difficult to estimate beforehand the impact of the study, we hope to encourage open science by protocol publication and sharing extraction sheets and codes to inspire future research teams which could continue the examination of SFBT in the clinical setting by using our data extraction codebook to build pooled datasets. We hope our results will be of a practical use to the clinical practice and policy/commissioning with aligned economic endpoints (cost per remission, QALY) by providing moderator schema (dose, setting, delivery mode, provider characteristics), integrating the balance of benefits and potential harm, and highlighting also patient-important outcomes and goal-attainment for better clinical relevance.

### 4.1 Expected limitations and implications for future research

Several issues posing potential limitations are expected to be encountered during the realization of this project. An insufficient number of high-qualitative studies will decrease the confidence in findings. A low representation of the diversity of CMD will limit the understanding of SFBT efficacy and effectiveness in the clinical setting. Small sample size will impact the reliability of the results. Monitorization period may be limited to the minimal requirement of at least 3 months (5) with insufficient information to identify long-term effects. All limitations found during the study will be highlighted alongside recommendations for future research with potential impact on further attempts at organizing the evidence of SFBT efficacy and effectiveness in the clinical setting.

## 5 Dissemination

Findings will be submitted to peer-reviewed journals and presented at conferences on psychology and psychotherapy. A summary will also be prepared for practitioners and policy makers to provide empirical support regarding the evidence-based recommendation of SFBT for specific CMDs.
